# Indications of neoadjuvant chemotherapy for locally advanced Gastric Cancer patients based on pre-treatment clinicalpathological and laboratory parameters

**DOI:** 10.7150/jca.46430

**Published:** 2020-08-18

**Authors:** Yue Wang, Jun Zhang, Shuai Guo, Xiang-yu Meng, Zhi-chao Zheng, Yan Zhao

**Affiliations:** 1 Department of Gastric Cancer, Liaoning Cancer Hospital & Institute (Cancer Hospital of China Medical University), No. 44 Xiaoheyan Road, Dadong District, Shenyang City, Liaoning Province, China.; 2 China Medical University, No.77 Puhe Road, Shenyang North New Area, Shenyang, Liaoning Province, China.

**Keywords:** locally advanced gastric cancer, neoadjuvant chemotherapy, pre-treatment, indications, nomogram, recursive partitioning analysis

## Abstract

**Objective:** There are controversial indications for neoadjuvant chemotherapy (NAT) in the treatment of locally advanced gastric cancer (LAGC). Here, we aimed to identify indications for NAT based on pre-treatment clinicopathological and laboratory parameters.

**Methods:** This study included a retrospective cohort of 1083 LAGC patients who had underwent radical D2 gastrectomy in the Cancer Hospital of China Medical University between 2012 and 2016. After propensity score matching, 756 patients were recruited and were separated into NAT (n=378) or primary surgery (PS) (n=378) groups. Cox regression identified pre-treatment risk factors for overall survival (OS). A nomogram was established to predict OS and calculate scores for risk factors. Recursive partitioning analysis (RPA) determined cut off values, where the entire patient cohort was divided into low and high risk groups.

**Results:** Seven risk factors that were significantly related to OS were incorporated in the nomogram. These risk factors included age, tumor size, tumor site, carbohydrate antigen 199 (CA199), carcino-embryonic antigen (CEA), clinical T stage (cT) and clinical N stage (cN). The model contained a C-index of 0.637. The calibration curve revealed anticipated values that were reflective of actual values. The decision curve revealed an achievement of optimal clinical impact when threshold possibility was 0-54%. Next, the cohort was split into low (≤ 252 points) or high (> 252 points) risk groups based on the 5-year OS projected by RPA. The PS group showed a worse OS compared to the NAT group for high-risk patients (*P =*0.004). There was no significant difference when comparing OS between the PS and NAT groups for low-risk patients (*P =*0.407).

**Conclusions:** A feasible, quantifiable and practical prognostic tool was generated to screen for potential survival benefits for patients receiving NAT. Surgeons can use this model to identify optimal treatment regimens for individualized treatment strategies during the diagnosis of LAGC patients. For these patients, NAT is suggested for high-risk patients.

## Introduction

Gastric cancer (GC) is the fourth highest contributor of cancer-related deaths worldwide 1. Prognosis for GC is poor, where the 5-year overall survival (OS) rate ranges from only 25% to 31% 2-5. Due to high postoperative recurrence and metastasis rates in patients with locally advanced GC (LAGC), surgery alone remains unsatisfactory. There is a global consensus to combine surgery with chemotherapy and radiotherapy for a more comprehensive perioperative treatment strategy. As an important part of perioperative therapy, neoadjuvant chemotherapy (NAT) is recommended by various international guidelines, but there are still significant differences in patient selection algorithms. The 2019 National Comprehensive Cancer Network (NCCN) guidelines recommend that patients with a clinical TNM (cTNM) stage ≥ T2N should receive NAT 6. The 2016 European Society of Oncology (ESMO) guidelines recommend that patients with a cTNM stage > T1N0 should accept NAT 7. The fifth edition of Japanese Gastric Cancer Treatment Guidelines recommends that patients with a cTNM stage ranging from T2 to T4 and a bulky N should receive NAT 8. In China, the number of newly diagnosed GC cases account for 42.5% worldwide, where the percentage of LAGC cases account for 70% of these total cases 9. The 2018 Chinese Society of Clinical Oncology (CSCO) guidelines recommend that patients with a cTNM stage of cIII should accept NAT 10.

There is no worldwide consensus on which specific stages should receive NAT, especially when it comes to tailoring to the specific treatment given to each patient. Even if NAT could be used to treat specific stages of GC, not all patients would benefit from NAT as expected. Recently, nomograms have been used for tumor prognosis prediction models after NAT 11-16. These models show superior performance compared to conventional staging models 17-21. Most studies analyzing prognosis of individuals who underwent treatment and follow-ups use nomograms. However, there have only been a few reports of a prognosis prediction model associated with pre-treatment routine inspection factor nomograms of patients initially admitted for LAGC. A nomogram prediction model based on pre-treatment parameters may be a feasible, quantifiable and prediction reference for identifying patients that would respond best to NAT.

Here, we construct pre-treatment nomogram prediction models to screen for patients that may benefit most from NAT based on previous routine inspections of pre-treatment prognostic factors. In this study, indications for the rational application of NAT for individualized treatment are suggested.

## Materials and Methods

### Patients

We conducted a retrospective cohort of patients between January 2012 and December 2016 who were diagnosed as LAGC (We only included Siewert type II and III cancers in our study, while Siewert type I was excluded for the adenocarcinoma of esophagogastric junction) in Liaoning Cancer Hospital and Institute (Cancer Hospital of China Medical University). The inclusion criteria included: (1) diagnosis of gastric adenocarcinoma by histopathological examination; (2) older than 18 years and younger than 75 years; (3) with a World Health Organization performance status score of 0 or 1 according to Eastern Cooperative Oncology Group (ECOG); (4) no other primary tumor in the previous 5 years; (5) No distant metastasis (M0); (6) the D2 gastrectomy were performed, the examined lymph nodes ≥ 15. The exclusion criteria included: (1) patients with non-adenocarcinoma histology; (2) distant metastatic disease (M1); (3) treatment variables consisted of use of preoperative radiotherapy/targeted therapy or postoperative radiotherapy; (4) remnant gastric cancer; (5) patients receiving intraperitoneal chemotherapy or hyperthermic intraperitoneal chemotherapy; (6) no receipt of D2 gastrectomy or no operation, the examined lymph nodes < 15; (7) R1 (microscopically incomplete resection) or R2 (macroscopically incomplete resection) margin status; (8) ECOG performance status ≥ 2; (9) patients whose follow-up or perioperative death within 3 months.

### Follow-up and survival analyses

Survival data was obtained using patient records, outpatient services or phone interviews. The OS was measured based on the first day of NAT or surgery to death or final follow-up (June 2019). Patients excluded from this study included those who passed away from complications experienced during surgery, had follow-ups of 3 months or less or did not have strong follow-up records. The average follow-up time was 56 months (4.1-95.3 months).

### Perioperative chemotherapy

NAT was provided to patients for 2 to 6 cycles, with adjustments to dosage or cycles based on effectiveness and patient tolerability. Perioperative chemotherapeutics given to patients included oxaliplatin, capecitabine and tegafur gimeracil oteracil potassium capsules (SOX and XELOX regimens). Responses to NAT were analyzed based on the response of the primary tumor and whether it was shrinking as shown by endoscopy, ultrasound endoscopy and three-dimensional enhanced computed tomography. Patients underwent surgery 2 to 4 weeks after NAT. An upfront surgery followed by adjuvant treatment with 2 to 6 cycles of the same chemotherapeutic agents. Patients who received primary surgery, if there were no obvious contraindications, would undertake postoperative chemotherapy of either XELOX or SOX regimens for 6-8 cycles. For both NAT and primary surgery (PS) group, we recommended a total of 6-8 cycles of chemotherapy. The cycles of preoperative and postoperative chemotherapy were amounted in total planned cycles.

### Pre-treatment clinicopathological and laboratory parameter data collection

The pre-treatment clinicopathological and laboratory features analyzed in this study include age, gender, tumor size, tumor site, smoking history, drinking history, histological type, Borrmann type, neutrophil-to-lymphocyte ratios (NLR), platelet-to-lymphocyte ratios (PLR), carcino-embryonic antigen (CEA), carbohydrate antigen 199 (CA199), clinical T stage (cT) and clinical N stage (cN). Tumor size, Borrmann type and histological type were rigorously evaluated by two endoscopic physicians, radiologists and three independent pathologists. For a controversial diagnosis, they reviewed the cases by internal discussion until reaching an agreement. Preoperative T and N staging criteria were followed as described previously 22-25. Primary tumors were staged according to the 8th edition American Joint Committee on Cancer (AJCC). The 8th edition AJCC TNM were T2-T4NanyM0. (Figure S1). Informed consent was obtained for each patient. This study was approved by the Ethics Committee of Liaoning Cancer Hospital & Institute.

### Statistical analysis

To analyze the significance for measurement data, the Chi-square or Fisher's exact tests were used. To analyze enumeration data, t-tests or the Mann-Whitney U test were used. The Kaplan Meier method was used to generate and analyze survival curves whereas the log-rank test was performed to identify significant differences in survival. Cox regression was performed to identify independent risk factors influencing OS. Prognostic factors were analyzed with an adjusted hazard ration containing a 95% confidence interval. To predict OS, a nomogram was generated and was analyzed for accuracy based on Harrell's concordance index (C-index). Internal validation was conducted using a calibration curve and 1000 repeats. Plots were generated where solid lines indicated actual and dotted lines indicated expected values. The “X-tile” program was used to determine the cut-off values of the tumor size and pre-treatment NLR and PLR (Figure S2). The calculated cut-off value of tumor size was 4.8 cm, so we chose 5 cm as the cut-off in our study for application purpose. The most suitable cut-off value for the nomogram to calculate 5-year OS was determined using recursive partitioning analysis (RPA), which is a method that separates patients into different risk groups in order to identify survival predictors. R software ('rpart' package) uses an algorithm that works to select the predictor and provide an optimal division between two subgroups that were more similar in respect to outcome. In addition, each subgroup was further divided into groups by identifying a variable to split the group, which continued until there were too few values for more divisions. This pruning was used on the original partitioning tree to identify a point where there was a maximization of the predictive accuracy to prevent overfitting of the data. Decision curve analysis (DCA) analyzed clinical utility of the nonogram 26, 27. The y-axis represented net benefits and the x-axis measured threshold probability (Pt). The horizontal solid line indicated the advantage for patients not receiving NAT, the oblique solid line represented the advantage for patients receiving NAT and the diagonal dotted line (nomogram) indicated survival on the basis of nomogram scores to resolve whether a patient should receive NAT. Net benefit was calculated by subtracting relative harms (false positive) from benefits (true positive) 28, 29. A treatment strategy was superior if it had the highest value compared to other models, including two simple strategies, such as performing NAT for all patients (sloping solid line) or performing primary surgery first (horizontal solid line). SPSS version 24 (SPSS, Chicago, IL), Stata 15.1 (StataCorp, College Station, TX), X-tile, EmpowerStats software (EmpowerStats, X&Y Solutions, Boston, MA) as well as R version 3.5.1 were used for statistical analyses. A two-sided *p* < 0.05 was considered to be statistically significant.

## Results

### Patient Characteristics

A total of 1083 patients were enrolled in this study and 469 were given NAT followed by surgery and adjuvant chemotherapy (NAT group), while 614 received primary surgery followed by adjuvant chemotherapy (PS group) (Table S1).

Considering unmatched characteristics between the two groups based on retrospective analysis, a 1:1 case-control matched analysis for the NAT and PS groups was generated. After propensity score matching, a total of 756 individuals were placed in the NAT (n=378) and PS (n=378) groups for further evaluation (Table 1). No significant differences were identified when comparing all patient characteristics between the NAT and PS groups (*P* > 0.05). The completion of the planned chemotherapy tended to differ between the two groups, with more dosage adjustments needed in PS group. However, there were no significant differences between the two groups on the issue of chemotherapy planned or accomplished (Table S2).

### Nomogram calculating OS after 1:1 propensity score matching

**Figure 1:**
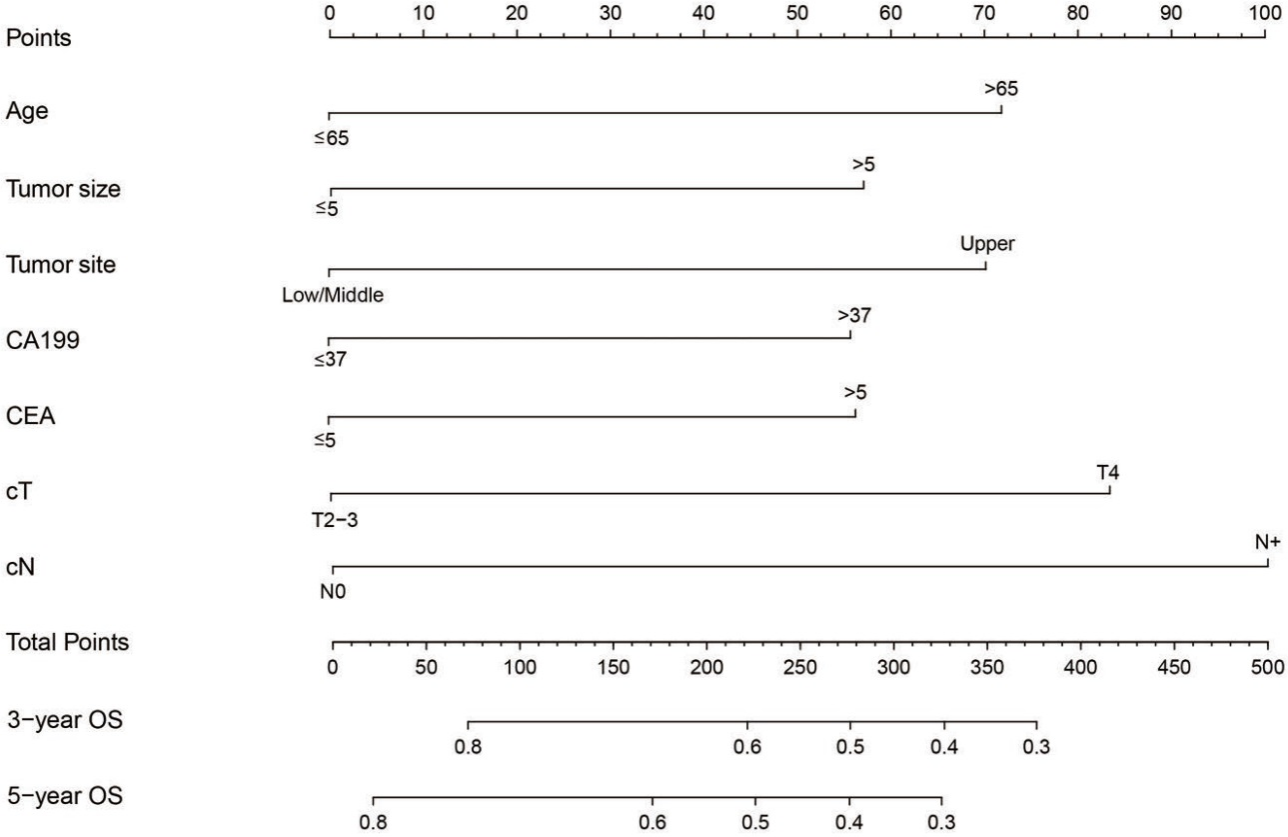
A nomogram predicting OS after a 1:1 propensity score matching 3 and 5 years before treatment. These probabilities are generated based on total points calculated as the sum for each specific variable.

Pre-treatment factors affecting OS were analyzed using univariate and multivariate cox proportional models. Gender, age, tumor size, tumor site, Borrmann type, CEA, CA199, PLR, cT and cN significantly influenced the OS based on univariate cox regression analysis. Age, tumor size, tumor site, CEA, CA199, cT and cN were found to be independent pre-treatment risk factors affecting prognosis based on multivariate cox regression analysis (Table 2). A nomogram prediction model integrating independent prognostic factors based on multivariate cox model was generated (Figure 1). Each risk factor included in the nomogram was assigned a number of points and the cut-off for risk factors influencing OS is shown in Table S3. Total points were generated based on the sum of each factor point value which was converted into 3 or 5 year OS to determine death probability for each patient. The C-index of the forecasting model for OS projection was 0.637. Calibration plots showed similarities with actual OS probabilities predicted from the nomogram (Figure S3).

### Optimal cut-off value for stratification by nomogram scores

**Figure 2:**
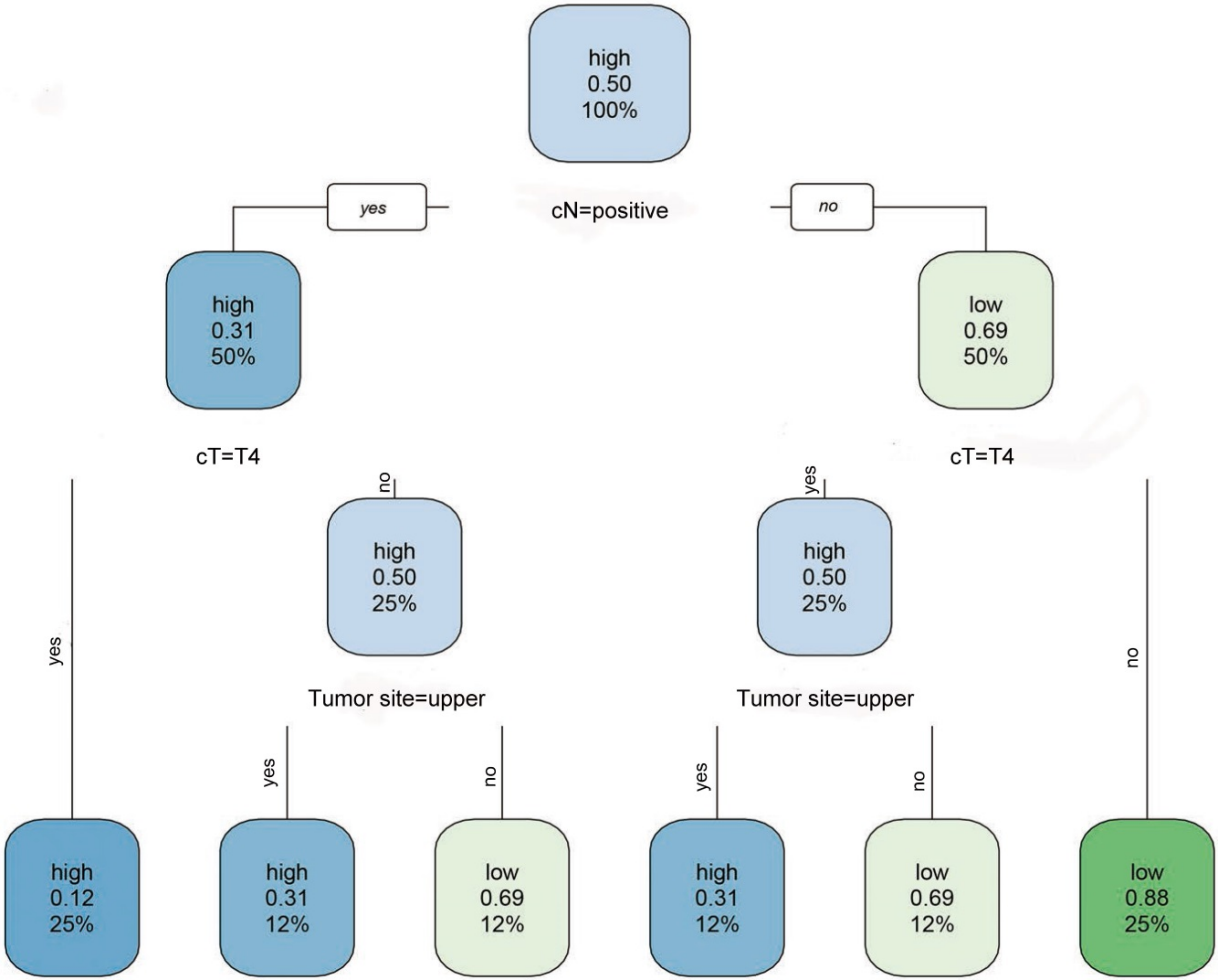
A flow chart for high and low-risk patients derived by recursive partitioning analysis (RPA). Risk presented in the node was identified using a green (low) to blue (high) gradient. The other value in the node is the predicted survival probability ranging from 0.12 to 0.88. The third value presented is the number of observations belonging to the group.

To confirm the optimal cut-off value used for stratification of the two populations, RPA was used. Based on RPA, the optimal cut-off value for 5-year OS as shown in the nomogram projection was 252. All 758 patients were reorganized into new groups based on cut-off values. Patients with total scores ≤ 252 were considered low-risk, while patients with total scores > 252 were defined as a high-risk group (Figure 2).

### Comparing OS in different risk groups

**Figure 3:**
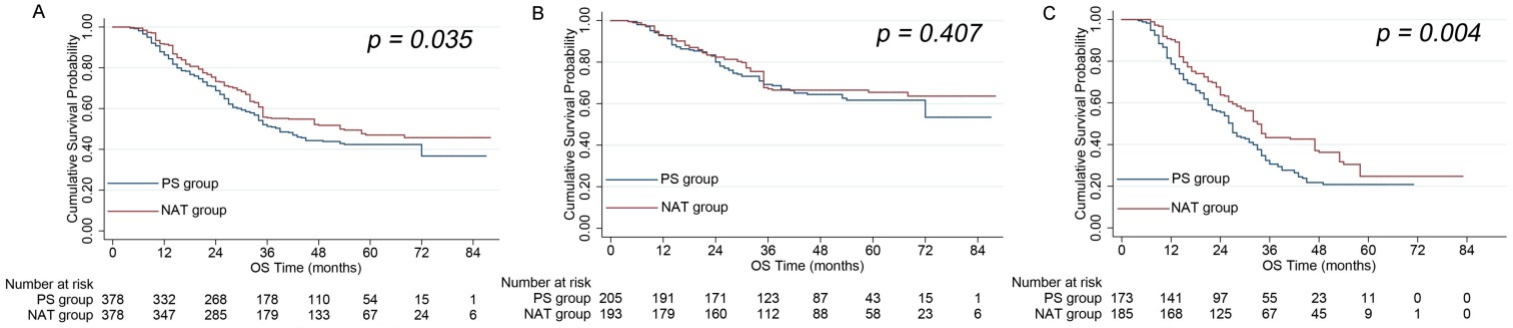
** A.** A comparison of OS between the NAT and PS groups for all patients. **B.** A comparison of OS between the NAT and PS groups for low-risk patients. **C.** A comparison of OS between the NAT and PS groups for high risk-patients.

In the NAT group, the 3 and 5 year OS rates were 68% and 49%, respectively. In the PS group, the 3 and 5 year OS rates were 63% and 41%, respectively. These data indicated that the NAT group showed significantly better OS than the PS group (*P =* 0.035) (Figure 3A). RPA stratification revealed that the NAT group still showed a stronger OS rate compared to the PS group when analyzing high-risk patients (*P =* 0.004) (Figure 3C). However, the NAT group showed a similar OS rate to the PS group when analyzing low-risk patients (*P =* 0.407) (Figure 3B).

### Clinical utility

**Figure 4:**
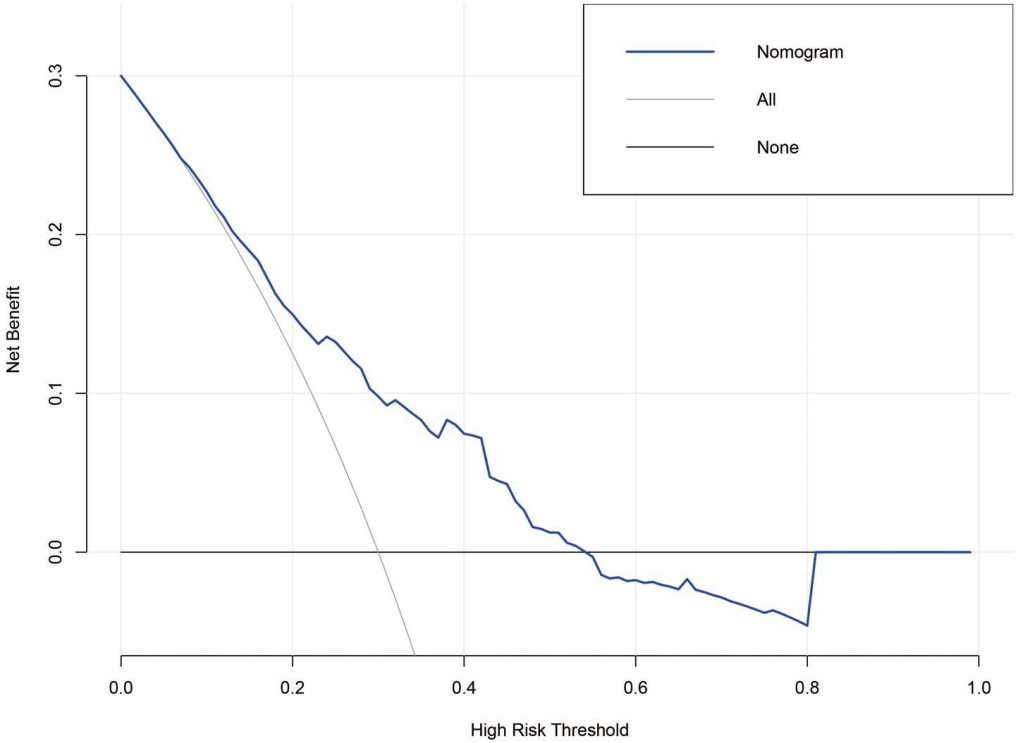
The DCA analyzed clinical utility of the nonogram. The y-axis represented net benefits and the x-axis measured threshold probability (Pt). The horizontal solid line indicated the advantage for patients not receiving NAT, the oblique solid line represented the advantage for patients receiving NAT and the diagonal dotted line (nomogram) indicated survival on the basis of nomogram scores to resolve whether a patient should receive NAT. A treatment strategy was superior if it had the highest value compared to other models, including two simple strategies, such as performing NAT for all patients (sloping solid line) or performing primary surgery first (horizontal solid line). For example, the value of net benefits would be 0.100 if we select 29% as cutoff value, which means that nomogram would find about 10 patients benefit from NAT among 100 patients compared with primary surgery.

The value of the nomogram and its use in the clinic was evaluated by DCA (Figure 4). Data demonstrated that a threshold possibility of 0-54% indicated that survival advantage of the diagonal dotted line (the NAT cohort based on nomogram score) was more greater than the horizontal solid line (the cohort that did not receive NAT) and the oblique solid line (the cohort that received NAT). Therefore, the population with the maximum advantage was determined, which indicated that NAT should be given to patients with risk scores > 252. Compared to cohorts were zero or all LAGC patients received NAT, this approach would gain maximum benefit for the NAT.

## Discussion

To explore the best treatment methods for LAGC that are used around the world, a series of clinical studies were performed 30-35. Based on clinical data for the perioperative treatment of patients that underwent D1 radical resection in Europe, MAGIC 30, FNCLCC&FFCD 31 and FLOT4 32, 33 studies confirmed that NAT + operation + adjuvant chemotherapy prevented progression and prolonged survival. However, patients selected for these studies were mainly non-Asian. Asian scholars successively performed studies related to perioperative chemotherapy for other schemes and mainly focused on D2 radical surgery. The ESMO annual meeting in 2019 announced two randomized controlled trials for perioperative chemotherapy in Asia including the PRODIGY study in South Korea and the RESOLVE study in China 34, 35. This provided strong evidence that perioperative chemotherapy methods were also suitable for LAGC treatment in Asian patients. At the same time, meta-analysis suggested that perioperative chemotherapy was superior to traditional adjuvant chemotherapy for LAGC cases 36-39. In our study, large retrospectively analyzed data was used through propensity score matching to further explore whether perioperative chemotherapy brought survival benefit to LAGC patients. Presently, there is a global consensus to combine NAT + surgery + adjuvant chemotherapy for comprehensive perioperative treatment against LAGC.

However, there is no global consensus on indications for NAT. This diversity stems not only from different treatment methods between different parts of the world but also based on the surgical approaches used between them. NAT was recommended for patients with > T1N0 who underwent D1 radical resection based on ESMO and NCCN guidelines 6, 7, making it difficult to directly apply to patients with LAGC who underwent D2 radical resection. Focused on the concept of D2 radical surgery, many Asian scholars explored indications for NAT. The results of the JCOG1302A study performed in Japan suggested that indication for NAT based on existing diagnostics may be restricted to advanced stages of cT3~4+cN1~3 cases 40. The PRODIGY study enrolled patients with LAGC or esophagogastric junction adenocarcinoma with a clinical stage of T2-3/N+M0 or T4/NanyM0 36. The clinical stage enrolled in the RESOLVE study in China was more advanced than the PRODIGY study (T4a/N+M0 or T4b/NanyM0) 37. All studies believed that NAT benefited certain subgroups rather than all patients in advanced stages. Our study provides a more thorough investigation and generation of a pre-treatment prediction model through a nomogram for LAGC patients. The goal of this was to identify a cut-off point distinguishing patients more likely to benefit from NAT. Based on these results, patients in the cTNM stage of T4N+M0 showed greatest indication for NAT under D2 radical surgery, which is consistent with other work 41.

Presently, NAT is given based on T and N stages. Other objective independent parameters may serve as additional indications for NAT selection. Other factors included upper GC, CEA > 5ng/mL, CA199 > 37U/mL, tumor size > 5cm and age > 65 years, which could be scored by a nomogram. According total scores for these factors including T and N stages, the cut off scores for receiving NAT was determined by RPA. Each patient could be provided a total score which indicated risk grade before identifying a treatment plan. Thus, one could accurately judge the pre-treatment prognosis of a patient. Additionally, this pre-treatment prediction model provides a feasible, quantifiable and practical prediction tool to distinguish between different patient risk groups. Furthermore, individualized treatment for newly diagnosed LAGC patients could be quickly generated not just based on T and N stages. With this, clinicians could have more accurate and focused pre-treatment discussions with high-risk patients. Data from the JCOG0405 42, JCOG1002 43 and JCOG0210 44 studied performed in Japan also showed that LAGC cases combined with swollen lymph nodes, a tumor diameter > 7 cm and linitis plastica greatly benefit from NAT. As for inflammation and immune related factors such as PLR and NLR, one group 45 also confirmed that elevated pre-treatment NLR levels showed value in survival prediction. One Germany study included a retrospective GC cohort of 410 patients revealed that tumor originated in the upper 2/3 part of the stomach tend to had a better response to NAT 46. A similar finding was also confirmed in Li. et al.'s study 47, which was consistent with our result. Some previous studies indicated that well differentiated tumors and intestinal type tumor by Lauren classification tend to benefit from NAT. It is widely accepted that Lauren classification was a useful indicator to predict chemotherapy response. However, it was practically hard to definite a Lauren classification by biopsy tissues, and the inter-tumor heterogeneity of stomach cancer might add complexity to the final analysis. We failed to show the correlation between chemotherapeutic response and any microscopic pathological classifications in the current study. However, it is reasonable to believe that pathological and molecular genetic or epigenetic markers and their combinations are potentially useable markers in predicting NAT response which need future investigation. Nevertheless, we obtained a set of pre-treatment factors that make individualized treatment practical.

As far as we know, this report describes the first and big data based study focused on whether NAT is requisite of LAGC using nomogram and RPA. There are certain limitations of this study. First, this study was conducted at a single center and the included patients only originated from Eastern countries which would cast doubts on its generalizability. Although single center study can control data quality and uniformity of surgical standard easily, external verification in non-Asia cohorts needs to be further validated. Second, the data regarding pre-treatment factors, such as body mass index, Lauren classification, lymphocyte-to-monocyte ratio, CA125, CA724, AFP, biomarkers (Her-2 and Ki-67 status), molecular typing (microsatellite and epstein barr virus status), were incomplete which could have biased the estimates of current study. Third, we only screen the potential beneficial patients receiving NAT. The beneficial patients of other combined treatment modes such as neoadjuvant chemoradiotherapy, neoadjuvant chemotherapy combined targeted therapy and/or immunotherapy are still unknown.

In conclusion, we have constructed a prognostic tool based on a nomogram and RPA to screen for potential survival benefits in LAGC patients receiving NAT. Clinicians can use this model to formulate individualized treatment strategies for LAGC patients. Patients in high risk may be more likely to benefit from NAT, these important issues would just be the triggers of yet randomized clinical trials to clarify in the future.

## Supplementary Material

Supplementary figures and tables.

## Figures and Tables

**Table 1:** Pre-treatment clinicalpathological and laboratory characteristics of LAGC with Group PS and Group NAT after 1:1 matched

Characteristics	Total	Group PS	Group NAT	*P* value
	n (%), (n=756)	n (%), (n=378)	n (%), (n=378)	
**Gender**				0.694
Female	233 (30.82%)	114 (30.16%)	119 (31.48%)	
Male	523 (69.18%)	264 (69.84%)	259 (68.52%)	
**Age (years)**				0.356
≤ 65	500 (66.14%)	256 (67.72%)	244 (64.55%)	
> 65	256 (33.86%)	122 (32.28%)	134 (35.45%)	
**Smoking history**				0.242
No	342 (43.24%)	179 (47.35%)	163 (43.12%)	
Yes	414 (54.76%)	199 (52.65%)	215 (56.88%)	
**Drinking history**				0.507
No	441 (58.33%)	225 (59.52%)	216 (57.14%)	
Yes	315 (41.67%)	153 (40.48%)	162 (42.86%)	
**Histologic type**				0.283
Other types of adenocarcinoma	598 (79.10%)	293 (77.51%)	305 (80.69%)	
Signet ring cell carcinoma	158 (20.90%)	85 (22.49%)	73 (19.31%)	
**Tumor site**				0.871
Lower/Middle	546 (72.22%)	274 (72.49%)	272 (71.96%)	
Upper	210 (27.78%)	104 (27.51%)	106 (28.04%)	
**Tumor size (cm)**				0.602
≤ 5	295 (36.02%)	151 (39.95%)	144 (38.10%)	
> 5	461 (60.98%)	227 (60.05%)	234 (61.90%)	
**NLR**				0.803
≤ 1.3	561 (74.21%)	279 (73.81%)	282 (74.60%)	
> 1.3	195 (25.79%)	99 (26.19%)	96 (25.60%)	
**PLR**				0.818
≤ 190.7	499 (66.00%)	248 (65.61%)	251 (66.40%)	
> 190.7	257 (34.00%)	130 (34.39%)	127 (33.60%)	
**CA199 (U/mL)**				0.348
≤ 37	649 (85.85%)	329 (87.04%)	320 (84.66%)	
> 37	107 (14.15%)	49 (12.96%)	58 (15.34%)	
**CEA (ng/mL)**				0.172
≤ 5	576 (76.19%)	296 (78.31%)	280 (74.07%)	
> 5	180 (23.81%)	82 (21.69%)	98 (25.93%)	
**Borrmann type**				0.156
I/II	293 (38.76%)	156 (41.27%)	137 (36.24%)	
III/IV	463 (61.24%)	222 (58.73%)	241 (63.76%)	
**Clinical T stage (cT)**				0.572
T2-3	138 (18.25%)	72 (19.05%)	66 (17.46%)	
T4	618 (81.75%)	306 (80.95%)	312 (82.54%)	
**Clinical N stage (cN)**				0.155
N0	114 (15.08%)	64 (16.93%)	50 (13.23%)	
N+	642 (84.92%)	314 (83.07%)	328 (86.77%)	

*P* values are marked in bold if less than 0.05; LAGC, locally advanced gastric cancer; PS, primary surgery; NAT, neoadjuvant chemotherapy; NLR, neutrophil-to-lymphocyte ratio; PLR, platelet-to-lymphocyte ratio; CA199, carbohydrate antigen 199; CEA, carcino-embryonic antigen.

**Table 2:** Univariate and multivariate cox regression analysis of OS for the whole LAGC patients after 1:1 matched

Variable	Univariate analysis		Multivariate analysis	
	HR (95% CI)	*P* value	HR (95% CI)	*P* value
**Gender**				
Female	Reference	-	Reference	-
Male	0.769 (0.626-0.946)	0.013	0.861 (0.694-1.068)	0.174
**Age (years)**				
≤ 65	Reference	-	Reference	-
> 65	1.556 (1.258-1.925)	<0.001	1.374 (1.104-1.710)	0.004
**Smoking history**				
No	Reference	-	Reference	-
Yes	0.982 (0.805-1.197)	0.854	-	-
**Drinking history**				
No	Reference	-	Reference	-
Yes	1.049 (0.859-1.282)	0.640	-	-
**Histologic type**				
Other types of adenocarcinoma	Reference	-	Reference	-
Signet ring cell carcinoma	0.877 (0.690-1.114)	0.281	-	-
**Tumor site**				
Lower/Middle	Reference	-	Reference	-
Upper	1.468 (1.198-1.798)	**<0.001**	1.486 (1.209-1.826)	**<0.001**
**Tumor size (cm)**				
≤ 5	Reference	-	Reference	-
> 5	1.850 (1.376-2.486)	**<0.001**	1.445 (1.066-1.958)	**0.018**
**NLR**				
≤ 1.3	Reference	-	Reference	-
> 1.3	0.858 (0.680-1.081)	0.194	-	-
**PLR**				
≤ 190.7	Reference	-	Reference	-
> 190.7	1.245 (1.015-1.528)	**0.035**	1.210 (0.982-1.490)	0.073
**CA199 (U/mL)**				
≤ 37	Reference	-	Reference	-
> 37	1.805 (1.411-2.307)	**<0.001**	1.317 (1.015-1.710)	**0.039**
**CEA (ng/mL)**				
≤ 5	Reference	-	Reference	-
> 5	1.612 (1.299-1.999)	**<0.001**	1.290 (1.026-1.623)	**0.029**
**Borrmann type**				
I/II	Reference	-	Reference	-
III/IV	1.373 (1.114-1.693)	**0.003**	1.107 (0.887-1.382)	0.369
**Clinical T stage (cT)**				
T2-3	Reference	-	Reference	-
T4	1.721 (1.399-2.117)	**<0.001**	1.549 (1.246-1.926)	**<0.001**
**Clinical N stage (cN)**				
**N0**	Reference	-	Reference	-
**N+**	2.163 (1.514-3.091)	**<0.001**	1.752 (1.215-2.525)	**0.003**

*P* values are marked in bold if less than 0.05; OS, overall survival; LAGC, locally advanced gastric cancer; HR, hazard ratios; CI, confidence interval; NLR, neutrophil-to-lymphocyte ratio; PLR, platelet-to-lymphocyte ratio; CA199, carbohydrate antigen 199; CEA, carcino-embryonic antigen.

## Abbreviations

GC: gastric cancer
NAT: neoadjuvant chemotherapy
LAGC: locally advanced gastric cancer
PS: primary surgery
OS: overall survival
DCA: decision curve analysis
RPA: recursive partitioning analysis
CA199: carbohydrate antigen 199
CEA: carcino-embryonic antigen
cT: clinical T stage
cN: clinical N stage
cTNM: clinical TNM stage
NCCN: National Comprehensive Cancer Network
ESMO: European Society of Oncology
CSCO: Chinese Society of Clinical Oncology
ECOG: Eastern Cooperative Oncology Group
NLR: neutrophil-to-lymphocyte ratios
PLR: platelet-to-lymphocyte ratios
AJCC: American Joint Committee on Cancer
C-index: concordance index

## References

[1] Siegel RL, Miller KD, Jemal A (2017). Cancer Statistics, 2017. CA Cancer J Clin.

[2] De Angelis R, Sant M, Coleman MP, Francisci S, Baili P, Pierannunzio D (2014). Cancer survival in Europe 1999-2007 by country and age: results of EUROCARE-5-a population-based study. Lancet Oncol.

[3] D'Angelica M, Gonen M, Brennan MF, Turnbull AD, Bains M, Karpeh MS (2004). Patterns of initial recurrence in completely resected gastric adenocarcinoma. Ann Surg.

[4] Ajani JA, D'Amico TA, Almhanna K, Bentrem DJ, Chao J, Das P (2016). Gastric Cancer, Version 3.2016, NCCN Clinical Practice Guidelines in Oncology. J Natl Compr Canc Netw.

[5] Allemani C, Matsuda T, Di Carlo V, Harewood R, Matz M, Nikšić M (2018). Global surveillance of trends in cancer survival 2000-14 (CONCORD-3): analysis of individual records for 37 513 025 patients diagnosed with one of 18 cancers from 322 population-based registries in 71 countries. Lancet.

[6] National Comprehensive Cancer Network. Clinical practice guidelines in oncology - gastric cancer: Gastric Cancer (2019.V1) [EB/OL] [2019 - 03 -14]

[7] Waddell T, Verheij M, Allum W, Cunningham D, Cervantes A, Arnold D (2014). Gastric cancer: ESMO-ESSO-ESTRO Clinical Practice Guidelines for diagnosis, treatment and follow-up. Radiother Oncol.

[8] Japanese gastric cancer treatment guidelines 2018 (5th edition) Gastric Cancer. 2020.

[9] Chen W, Zheng R, Zhang S, Zeng H, Xia C, Zuo T (2017). Cancer incidence and mortality in China, 2013. Cancer Lett.

[10] Wang FH, Shen L, Li J, Zhou ZW, Liang H, Zhang XT (2019). The Chinese Society of Clinical Oncology (CSCO): clinical guidelines for the diagnosis and treatment of gastric cancer. Cancer Commun (Lond).

[11] Lai J, Pan Z, Chen P, Ye G, Chen K, Su F (2019). Development and validation of a nomogram incorporating axillary lymph node ratio to predict survival in node-positive breast cancer patients after neoadjuvant chemotherapy. Jpn J Clin Oncol.

[12] Lai J, Wang H, Peng J, Chen P, Pan Z (2018). Establishment and external validation of a prognostic model for predicting disease-free survival and risk stratification in breast cancer patients treated with neoadjuvant chemotherapy. Cancer Manag Res.

[13] Kim CH, Yeom SS, Lee SY, Kim HR, Kim YJ, Lee KH (2019). Prognostic Impact of Perineural Invasion in Rectal Cancer After Neoadjuvant Chemoradiotherapy. World J Surg.

[14] Kwee RM, Kwee TC (2014). Role of imaging in predicting response to neoadjuvant chemotherapy in gastric cancer. World J Gastroenterol.

[15] Tan W, Yang M, Yang H, Zhou F, Shen W (2018). Predicting the response to neoadjuvant therapy for early-stage breast cancer: tumor-, blood-, and imaging-related biomarkers. Cancer Manag Res.

[16] Zhu YL, Sun YK, Xue XM, Yue JY, Yang L, Xue LY (2019). Unnecessity of lymph node regression evaluation for predicting gastric adenocarcinoma outcome after neoadjuvant chemotherapy. World J Gastrointest Oncol.

[17] Lin JX, Wang ZK, Wang W, Xie JW, Wang JB, Lu J (2019). Development and validation of a new staging system for node-negative gastric cancer based on recursive partitioning analysis: An international multi-institutional study. Cancer Med.

[18] Lin JX, Yoon C, Desiderio J, Yi BC, Li P, Zheng CH (2019). Development and validation of a staging system for gastric adenocarcinoma after neoadjuvant chemotherapy and gastrectomy with D2 lymphadenectomy. Br J Surg.

[19] Yuan SQ, Wu WJ, Qiu MZ, Wang ZX, Yang LP, Jin Y (2017). Development and Validation of a Nomogram to Predict the Benefit of Adjuvant Radiotherapy for Patients with Resected Gastric Cancer. J Cancer.

[20] Zheng ZF, Lu J, Wang W, Desiderio J, Li P, Xie JW (2018). Development and External Validation of a Simplified Nomogram Predicting Individual Survival After R0 Resection for Gastric Cancer: An International, Multicenter Study. Ann Surg Oncol.

[21] Li Z, Wang Y, Shan F, Ying X, Wu Z, Xue K (2018). ypTNM staging after neoadjuvant chemotherapy in the Chinese gastric cancer population: an evaluation on the prognostic value of the AJCC eighth edition cancer staging system. Gastric Cancer.

[22] Edge SB, Compton CC (2010). The American Joint Committee on Cancer: the 7th edition of the AJCC cancer staging manual and the future of TNM. Ann Surg Oncol.

[23] Kim HJ, Kim AY, Oh ST, Kim JS, Kim KW, Kim PN (2005). Gastric cancer staging at multi-detector row CT gastrography: comparison of transverse and volumetric CT scanning. Radiology.

[24] Dorfman RE, Alpern MB, Gross BH, Sandler MA (1991). Upper abdominal lymph nodes: criteria for normal size determined with CT. Radiology.

[25] Yang DM, Kim HC, Jin W, Ryu CW, Kang JH, Park CH (2007). 64 multidetector-row computed tomography for preoperative evaluation of gastric cancer: histological correlation. J Comput Assist Tomogr.

[26] Kerr KF, Brown MD, Zhu K, Janes H (2016). Assessing the Clinical Impact of Risk Prediction Models With Decision Curves: Guidance for Correct Interpretation and Appropriate Use. J Clin Oncol.

[27] Vickers AJ, Cronin AM, Elkin EB, Gonen M (2008). Extensions to decision curve analysis, a novel method for evaluating diagnostic tests, prediction models and molecular markers. BMC Med Inform Decis Mak.

[28] Vickers AJ, Elkin EB (2006). Decision curve analysis: a novel method for evaluating prediction models. Med Decis Making.

[29] Talluri R, Shete S (2016). Using the weighted area under the net benefit curve for decision curve analysis. BMC Med Inform Decis Mak.

[30] Cunningham D, Allum WH, Stenning SP, Thompson JN, Van de Velde CJ, Nicolson M (2006). Perioperative chemotherapy versus surgery alone for resectable gastroesophageal cancer. N Engl J Med.

[31] Ychou M, Boige V, Pignon JP, Conroy T, Bouché O, Lebreton G (2011). Perioperative chemotherapy compared with surgery alone for resectable gastroesophageal adenocarcinoma: an FNCLCC and FFCD multicenter phase III trial. J Clin Oncol.

[32] Al-Batran SE, Hofheinz RD, Pauligk C, Kopp HG, Haag GM, Luley KB (2016). Histopathological regression after neoadjuvant docetaxel, oxaliplatin, fluorouracil, and leucovorin versus epirubicin, cisplatin, and fluorouracil or capecitabine in patients with resectable gastric or gastro-oesophageal junction adenocarcinoma (FLOT4-AIO): results from the phase 2 part of a multicentre, open-label, randomised phase 2/3 trial. Lancet Oncol.

[33] Al-Batran SE, Homann N, Pauligk C, Goetze TO, Meiler J, Kasper S (2019). Perioperative chemotherapy with fluorouracil plus leucovorin, oxaliplatin, and docetaxel versus fluorouracil or capecitabine plus cisplatin and epirubicin for locally advanced, resectable gastric or gastro-oesophageal junction adenocarcinoma (FLOT4): a randomised, phase 2/3 trial. Lancet.

[34] Kang YK, Yook JH, Park YK, Kim YW, Kim j, Ryu mh (2019). LBA41 Phase III randomized study of neoadjuvant chemotherapy (CT) with docetaxel (D), oxaliplatin (O) and S-1 (S) (DOS) followed by surgery and adjuvant S-1, vs surgery and adjuvant S-1, for resectable advanced gastric cancer (GC) (PRODIGY). Ann Oncol.

[35] Ji JF, Shen L, Liang H, Xue YW, Zhang XT, Wang YN (2019). LBA42 Perioperative chemotherapy of oxaliplatin combined with S-1 (SOX) versus postoperative chemotherapy of SOX or oxaliplatin with capecitabine (XELOX) in locally advanced gastric adenocarcinoma with D2 gastrectomy: a randomized phase III trial (RESOLVE trial). Ann Oncol.

[36] Long B, Yu ZY, Li Q, Du HR, Wang ZJ, Zhan H (2018). A systematic review and network meta-analysis protocol of neoadjuvant treatments for patients with gastric cancer. Medicine (Baltimore).

[37] Yang Y, Yin X, Sheng L, Xu S, Dong L, Liu L (2015). Perioperative chemotherapy more of a benefit for overall survival than adjuvant chemotherapy for operable gastric cancer: an updated Meta-analysis. Sci Rep.

[38] Cai Z, Yin Y, Shen C, Wang J, Yin X, Chen Z (2018). Comparative effectiveness of preoperative, postoperative and perioperative treatments for resectable gastric cancer: A network meta-analysis of the literature from the past 20 years. Surg Oncol.

[39] Miao ZF, Liu XY, Wang ZN, Zhao TT, Xu YY, Song YX (2018). Effect of neoadjuvant chemotherapy in patients with gastric cancer: a PRISMA-compliant systematic review and meta-analysis. BMC Cancer.

[40] Fukagawa T, Katai H, Mizusawa J, Nakamura K, Sano T, Terashima M (2018). A prospective multi-institutional validity study to evaluate the accuracy of clinical diagnosis of pathological stage III gastric cancer (JCOG1302A). Gastric Cancer.

[41] Wang YK, Wang YC, Shan F, Tang L, Li ZY, Ji JF (2020). [Exploration of potential beneficial people of neoadjuvant chemotherapy based on clinical staging in gastric cancer: a single center retrospective study]. Zhonghua Wei Chang Wai Ke Za Zhi.

[42] Tsuburaya A, Mizusawa J, Tanaka Y, Fukushima N, Nashimoto A, Sasako M (2014). Neoadjuvant chemotherapy with S-1 and cisplatin followed by D2 gastrectomy with para-aortic lymph node dissection for gastric cancer with extensive lymph node metastasis. Br J Surg.

[43] Ito S, Sano T, Mizusawa J, Takahari D, Katayama H, Katai H (2017). A phase II study of preoperative chemotherapy with docetaxel, cisplatin, and S-1 followed by gastrectomy with D2 plus para-aortic lymph node dissection for gastric cancer with extensive lymph node metastasis: JCOG1002. Gastric Cancer.

[44] Iwasaki Y, Sasako M, Yamamoto S, Nakamura K, Sano T, Katai H (2013). Phase II study of preoperative chemotherapy with S-1 and cisplatin followed by gastrectomy for clinically resectable type 4 and large type 3 gastric cancers (JCOG0210). J Surg Oncol.

[45] Li Z, Li S, Ying X, Zhang L, Shan F, Jia Y (2020). The clinical value and usage of inflammatory and nutritional markers in survival prediction for gastric cancer patients with neoadjuvant chemotherapy and D2 lymphadenectomy. Gastric Cancer.

[46] Lorenzen S, Blank S, Lordick F, Siewert JR, Ott K (2012). Prediction of response and prognosis by a score including only pretherapeutic parameters in 410 neoadjuvant treated gastric cancer patients. Ann Surg Oncol.

[47] Li ZY, Koh CE, Bu ZD, Wu AW, Zhang LH, Wu XJ (2012). Neoadjuvant chemotherapy with FOLFOX: improved outcomes in Chinese patients with locally advanced gastric cancer. J Surg Oncol.

