# Supplementary material for: Indications of neoadjuvant chemotherapy for locally advanced Gastric Cancer patients based on pre-treatment clinicalpathological and laboratory parameters
Source: J Cancer. 2020 Aug 18;11(20):6000–8. doi: 10.7150/jca.46430 (PMC7477425; doi:10.7150/jca.46430)
Supplement: Supplementary file 1 — Supplementary figures and tables. [file jcav11p6000s1.pdf]

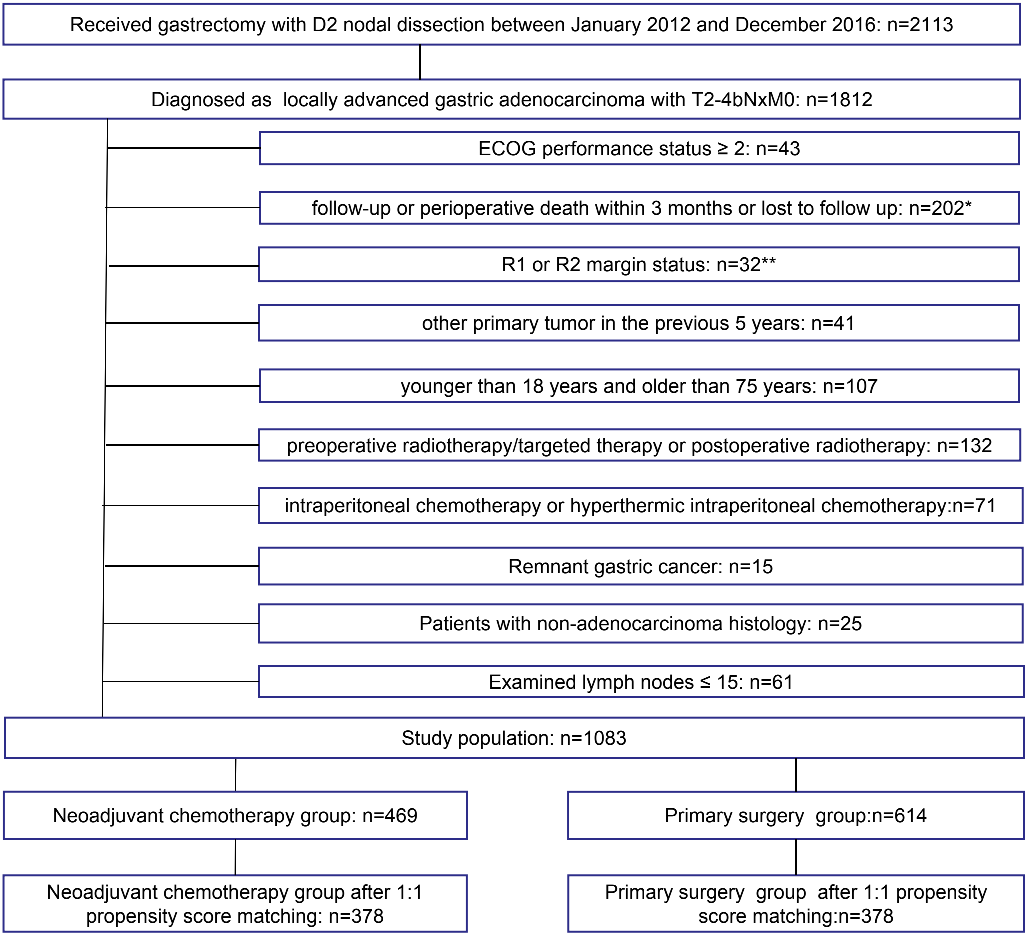

* Two patients encountered tumor progression during neoadjuvant chemotherapy and were transferred to secondly chemotherapy. Four patients gave up surgery after completion of NAC. The early stage death was 3 in NAT group and 2 in PS group, due to operative complications. Postoperative tumor progression accounted for 3 and 2 lethal events. We lost follow-up of 186 patients in our dataset.

**

--	NAT group	--	PS group	--	*P* value
R0	378	96.43%	378	95.45%	0.696#
R1	5	1.28%	8	2.02%	0.223##
R2	9	2.29%	10	2.53%	

Fisher exact test of n*m.

Fisher exact test of R1 vs. R2 in NAT and PS group.

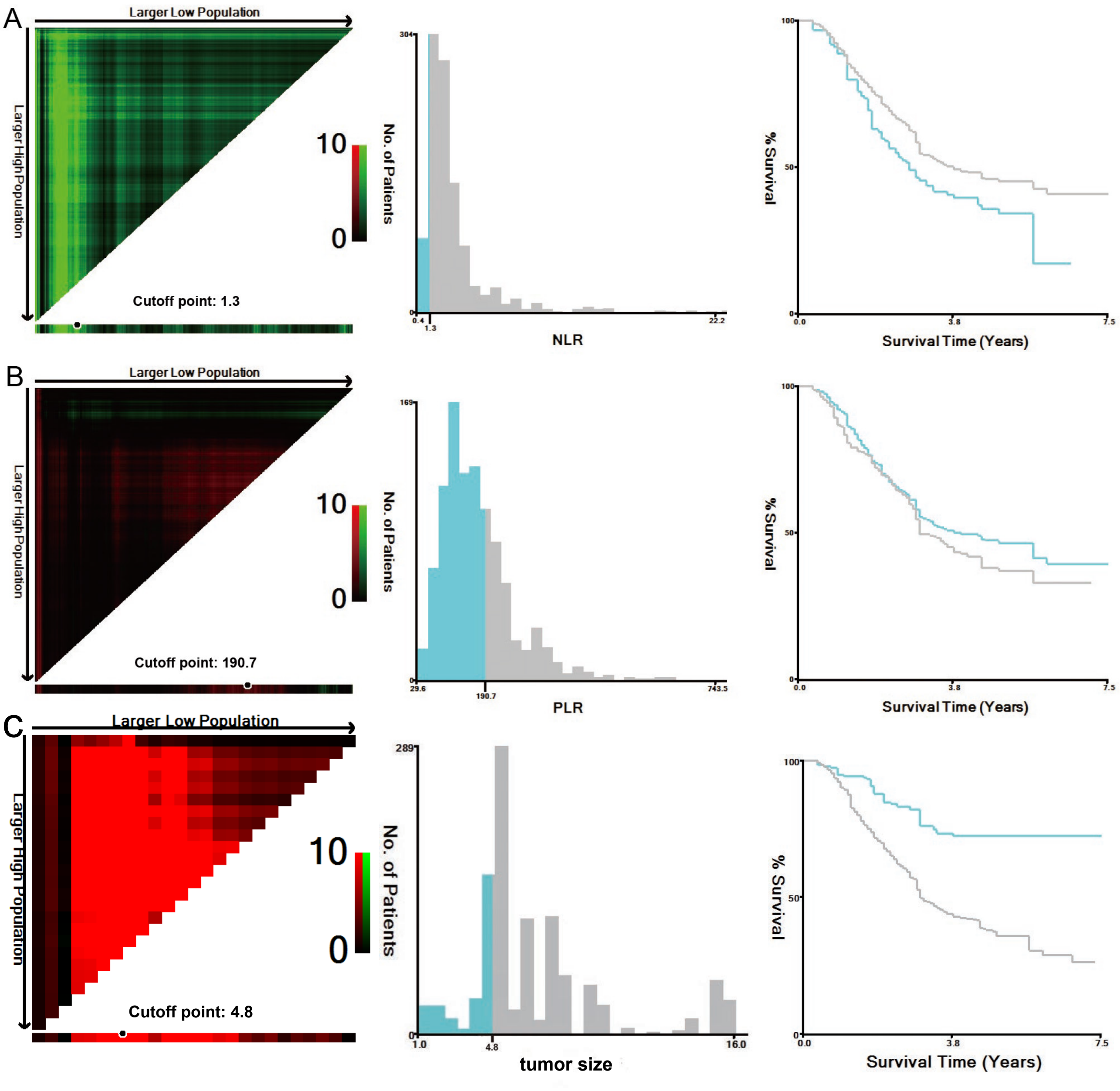

A
Larger Low Population
Larger High Population
10
0
Cutoff point: 1.3
No. of Patients
304
0
0.4
1.3
22.2
NLR
% Survival
100
50
0
0.0
3.8
7.5
Survival Time (Years)
B
Larger Low Population
Larger High Population
10
0
Cutoff point: 190.7
No. of Patients
169
0
29.6
190.7
743.5
PLR
% Survival
100
50
0
0.0
3.8
7.5
Survival Time (Years)
C
Larger Low Population
Larger High Population
10
0
Cutoff point: 4.8
No. of Patients
289
0
1.0
4.8
16.0
tumor size
% Survival
100
50
0
0.0
3.8
7.5
Survival Time (Years)

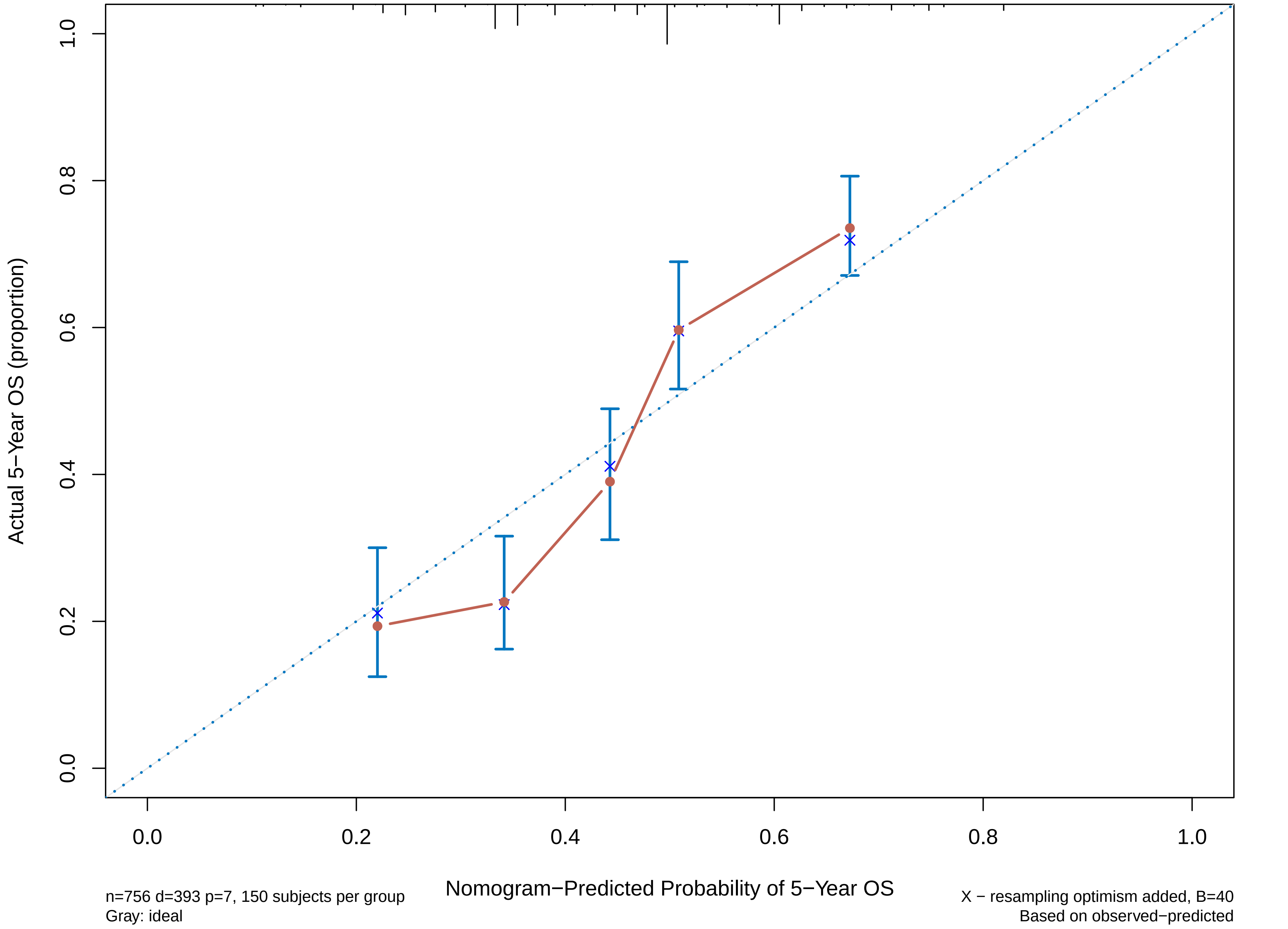

Actual 5−Year OS (proportion)
0.0
0.2
0.4
0.6
0.8
1.0
Nomogram−Predicted Probability of 5−Year OS
n=756 d=393 p=7, 150 subjects per group
Gray: ideal
X − resampling optimism added, B=40
Based on observed−predicted

Supplement table 1. Pre-treatment clinical characteristics of LAGC with Group PS and Group NAT before 1:1 matched

Characteristic	Total (n=1083) n (%)	Group PS (n=614) n (%)	Group NAT (n=469) n (%)	*P* value
**Gender**				**0.021**
Female	334(30.84%)	172(28.01%)	162(34.54%)	
Male	749(69.16%)	442(71.99%)	307(65.46%)	
**Age (years)**				0.594
≤ 65	697(64.36%)	391(63.68%)	306(65.25%)	
> 65	386(35.64%)	223(36.32%)	163(34.75%)	
**Smoking history**				0.365
No	495(45.71%)	288(46.91%)	207(44.14%)	
Yes	588(54.29%)	326(53.09%)	262(55.86%)	
**Drinking history**				0.616
No	642(59.28%)	368(59.93%)	274(58.42%)	
Yes	441(40.72%)	246(40.07%)	195(41.58%)	
**Histologic type**				0.152
Other types of adenocarcinoma	834(77.01%)	463(75.41%)	371(79.10%)	
Signet ring cell carcinoma	249(22.99%)	151(24.59%)	98(20.90%)	
**Tumor site**				0.841
Lower/Middle	703(64.91%)	397(64.66.%)	306(65.25%)	
Upper	380(35.09%)	217(35.34%)	163(34.75%)	
**Tumor size (cm)**				0.157
≤ 5	486(44.88%)	287(46.74%)	199(42.43%)	
> 5	597(55.12%)	327(53.26%)	270(57.57%)	
**NLR**				0.055
≤ 1.3	269(24.84%)	166(27.04%)	103(21.96%)	

> 1.3	814(75.16%)	448(72.96%)	366(78.04%)	
**PLR**				0.489
≤ 190.7	750(69.25%)	420(68.40%)	330(70.36%)	
> 190.7	333(30.75%)	194(31.60%)	139(29.64%)	
**CA199 (U/mL)**				**0.002**
≤ 37	890(82.18%)	524(85.34%)	366(78.04%)	
> 37	193(17.82%)	90(14.66%)	103(21.96%)	
**CEA (ng/mL)**				0.519
≤ 5	789(72.85%)	452(73.62%)	337(71.86%)	
> 5	294(27.15%)	162(26.38%)	132(28.14%)	
**Borrmann type**				**0.015**
I/II	461(42.57%)	281(45.77%)	180(38.38%)	
III/IV	622(57.43%)	333(54.23%)	289(61.62%)	
**Clinical T stage (cT)**				**<0.001**
T2-3	237(21.88%)	158(25.73%)	79(16.84%)	
T4	846(78.12%)	456(74.27%)	390(83.16%)	
**Clinical N stage (cN)**				**0.012**
**N0**	208(19.21%)	134(21.82%)	74(15.79%)	
**N+**	875(80.79%)	480(78.18%)	395(84.22%)	

P values are marked in bold if less than 0.05

LAGC, locally advanced gastric cancer; PS, primary surgery; NAT, neoadjuvant chemotherapy; NLR, neutrophil-to-lymphocyte ratio; PLR, platelet-to-lymphocyte ratio; CA199, carbohydrate antigen 199; CEA, carcino-embryonic antigen.

Table S2. The chemotherapeutic cycles finished between the NAT group and PS group.

--	NAT group	(%)	PS group	(%)	*P* value
**Number of patients**	378		378		
**Full cycles without adjustment**	174	46.03	162	42.86	0.411 [a]
**Full cycles with adjustment**	90	23.81	81	21.43	
**Unfinished but more than 1/2 planned cycles**	72	19.05	89	23.54	
**Less than 1/2 planned cycles or denied**	42	11.11	46	12.17	
**Total number of chemotherapy cycles finished**	2503		2417		
**The total number of full chemotherapy cycles finished without adjustment**	1350	53.94	1260	52.13	0.167 [b]
**The total number of full chemotherapy cycles finished with adjustment**	676	27.01	634	26.23	
**The total number of more than 1/2 planned chemotherapy cycles finished although not finished full cycles**	356	14.22	391	16.18	
**The total number of less than 1/2 planned chemotherapy cycles finished**	121	4.83	132	5.46	

a: The Chi-square test was used to compare the number of chemotherapy finished patients by different scales as well as dosage adjustments between the two groups.

b: The Chi-square test was used to compare the total number of chemotherapy cycles finished by different scales as well as dosage adjustments between the two groups.

Supplement table 3. The point values for risk factors affecting OS.

Number	Factors	Points
**1**	**Age**	
	≤ 65	0
	> 65	72
**2**	**Tumor size**	
	≤ 5	0
	> 5	57
**3**	**Tumor site**	
	low/middle	0
	upper	70
**4**	**CA199**	
	≤ 37	0
	> 37	56
**5**	**CEA**	
	≤ 5	0
	> 5	56
**6**	**cT**	
	T2-T3	0
	T4	83
**7**	**cN**	
	N0	0
	N+	100

Figure legends and table captions

Figure S1: Flow chart depicting the patients selection process.

Figure S2:X-tile used to confirm the cutoff values for NLR (A), PLR (B) and tumor size (C).

Figure S3: A calibration curve predicting the probability for 5 year OS plotted on the x-axis and the actual OS rate plotted on the y-axis. Calibration plots agreed with bias corrected prediction and the ideal reference line containing 1000 additional bootstraps.

Table S1. Pre-treatment clinical characteristics of LAGC with Group PS and Group NAT before 1:1 matched

Table S2. The chemotherapeutic cycles finished between the NAT group and PS group.

Table S3. The point values for risk factors affecting OS.
